# Supplementary figures and images for: PRDM9 activity depends on HELLS and promotes local 5-hydroxymethylcytosine enrichment
Source: eLife. 2020 Oct 13;9:e57117. doi: 10.7554/eLife.57117 (PMC7599071; doi:10.7554/eLife.57117)

Table 1- source data file 1

**A**

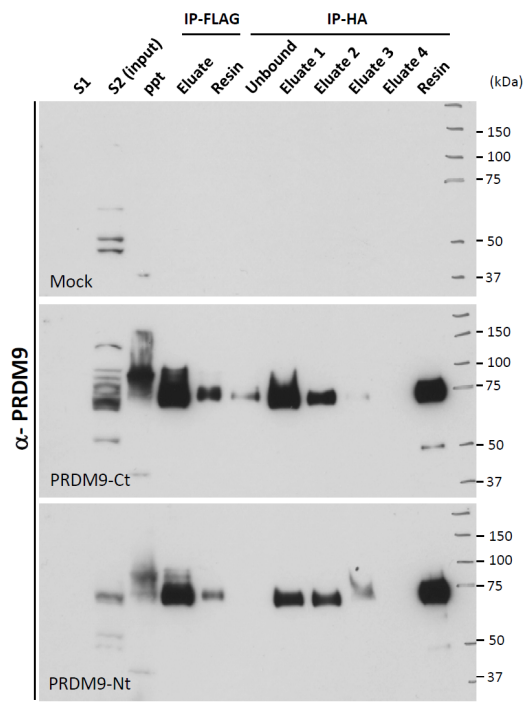

**B**

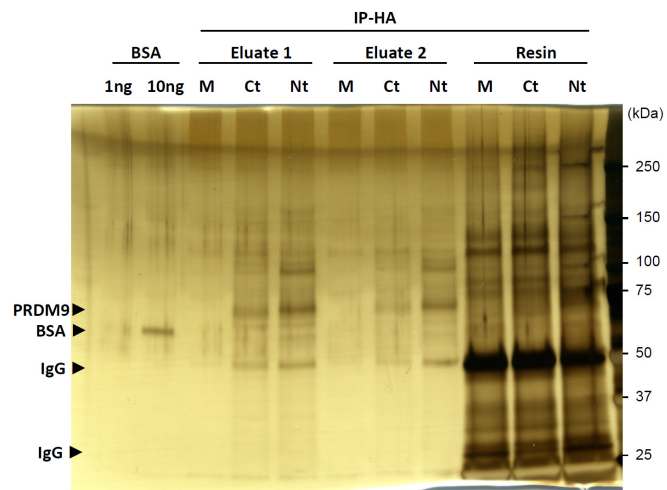

**C**

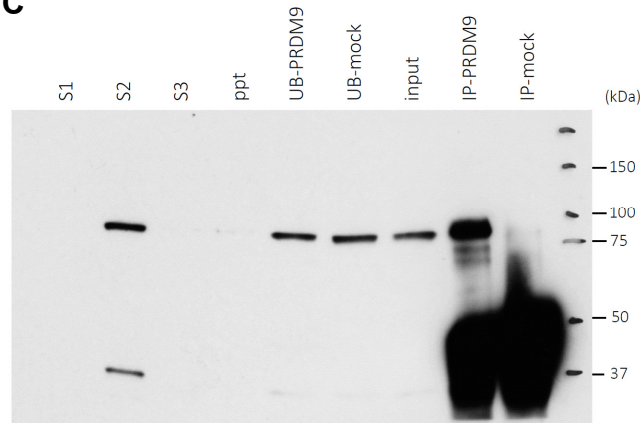

**D**

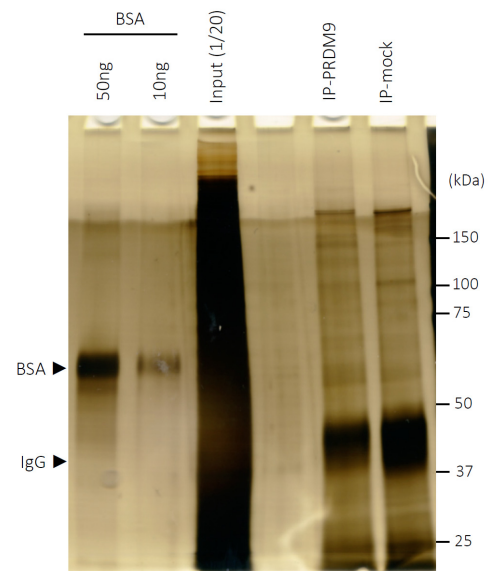

**E**

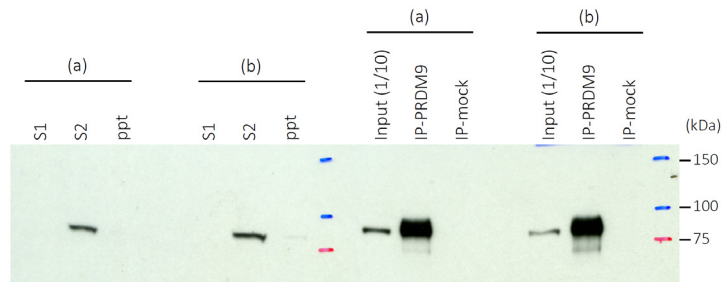

**F**

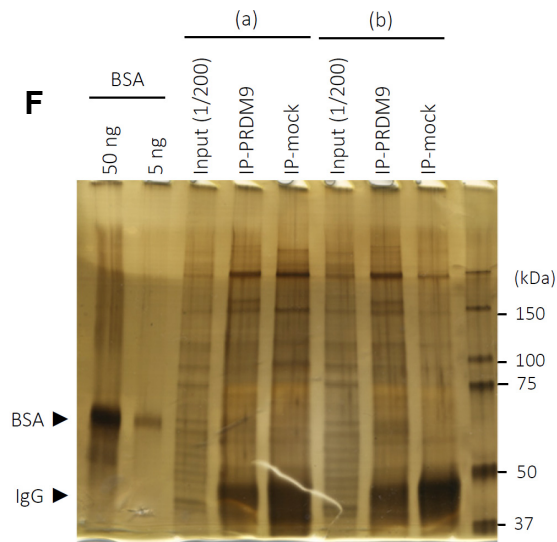

Supplement: Table 1—source data 1. — (A) Western blot analysis after complex purification by Flag-HA of extracts from HeLa S3 cells. HeLa S3 cells without PRDM9 expression vector, or expressing human PRDM9 tagged with Flag-HA at the C-terminus (PRDM9-Ct) or N-terminus (PRDM9-Nt) were used. Protein fractions of the extracts before IP (S1: cytoplasmic fraction, S2: nuclear fraction as input for IPs, ppt: insoluble pellet) and after the affinity purification steps were analyzed by western blotting using an anti-PRDM9 antibody. (B) Analysis of affinity-purified proteins after silver staining (sample without size selection). Eluates 1, 2 and resin fractions obtained from affinity purification (HA) of extracts initially prepared from HeLa S3 cells without PRDM9 expression vector (M), or expressing human PRDM9 tagged with Flag-HA at the C-terminus (Ct) or at the N-terminus (Nt) were separated by electrophoresis and silver stained. Mixtures of Eluate 1 and 2 were used for mass spectrometry analysis. (C) Western blot analysis of complex purification using an anti-PRDM9 antibody and mouse testes extracts (Mouse testis rep1). Protein extracts obtained during the Dignam-based purification (S1: cytoplasmic fraction, S2: nuclear fraction, S3: DNase-treated, and ppt: pellet) were loaded. Input (S2), unbound (UB), and proteins immunoprecipitated (IP) with an anti-PRDM9 antibody or normal rabbit serum (mock) were analyzed by western blotting. Detection was with an anti-PRDM9 antibody. Loading: 1 and 10% of input and IP samples, respectively. (D) Analysis of affinity-purified proteins by silver staining (Mouse testis rep1). Input, and samples IP with an anti-PRDM9 antibody or with normal rabbit serum (mock) were loaded. Bovine serum albumin (BSA) was used as control. Proteins were separated by electrophoresis and silver stained. (E) Western blot analysis of complex purification using an anti-PRDM9 antibody in extracts from mouse testes incubated with benzonase (Mouse testis rep 2), in duplicate (a and b). Protein extrac [file elife-57117-table1-data1.pdf]
