# Supplementary material for: PRDM9 activity depends on HELLS and promotes local 5-hydroxymethylcytosine enrichment
Source: eLife. 2020 Oct 13;9:e57117. doi: 10.7554/eLife.57117 (PMC7599071; doi:10.7554/eLife.57117)
Supplement: Supplementary file 2. [file elife-57117-supp2.docx]

**Supplementary File 2**

Primers used for genotyping *Hells* cKO mice

| **Name** | **Sequence** | **Genotype** | |  | **Annealing Temperature** | |
| --- | --- | --- | --- | --- | --- | --- |
| ***Hells 5’arm F1*** | TGTGCTTTGGACTAGAGATGTAGC |  | WT: 397bp  *Hells^fl^*: 559bp  *Hells^-^*: - | | 60°C | |
| ***Hells Ex12 R2*** | ACATTGTTCCAAAAATTCAAAAGG |  |  |  |  |  |
| ***Hells 5’arm F2*** | AGACCAAGCTGGCCTCAAACTC |  | WT, *Hells^fl^*: -  *Hells^-^*: 255bp | | 60°C | |
| ***Hells 3’arm R1*** | GTATGCACTAAAGAACGACAAGGA |  |  |  |  |  |
| ***Stra8 cnt FW*** | CTAGGCCACAGAATTGAAAGATCT |  | Control PCR: 324bp | | 60°C |  |
| ***Stra8 cnt RV*** | GTAGGTGGAAATTCTAGCATCATCC |  |  |  |  |  |
| ***Stra8 tg FW*** | GTGCAAGCTGAACAACAGGA |  | No transgene: -  *Stra8-Cre*: ~150bp | | 60°C |  |
| ***Stra8 tg RV*** | AGGGACACAGCATTGGAGTC |  |  |  |  |  |
