# Supplementary material for: PRDM9 activity depends on HELLS and promotes local 5-hydroxymethylcytosine enrichment
Source: eLife. 2020 Oct 13;9:e57117. doi: 10.7554/eLife.57117 (PMC7599071; doi:10.7554/eLife.57117)
Supplement: Supplementary file 3. [file elife-57117-supp3.docx]

**Supplementary File 3**

Primers used in RT-qPCR

| **Name** | **Sequence** | **Hotspot specificity** | |  | **Annealing Temperature** | |
| --- | --- | --- | --- | --- | --- | --- |
| ***Pbx1a-F*** | TAAATCATCGGGGCTTCC |  | *Dom2* | | 58°C | |
| ***Pbx1a-R*** | CCCCTTCCCCATAATACTGA |  |  |  |  |  |
| ***Chr.14a-F*** | TCCTGGTAATTTTTCTTGCTTT |  | *Dom2* | | 58°C | |
| ***Chr.14a-R*** | CCAATCCAATGTCCCTTACA |  |  |  |  |  |
| ***A3-F*** | TCTCAAACAATCCACACTGACC |  | *Dom2* | | | 58°C |
| ***A3-R*** | GGACAAGGCATGAGATAATCAA |  |  |  |  |  |
| ***Chr17b-F*** | CATGGACATGGAGACCTAACTG |  | *Dom2* | | | 58°C |
| ***Chr17b-R*** | TCAGTGGAAGCTCAGAAAATGA |  |  |  |  |  |
| ***Hlx1.6-F*** | TCCAGAGAAGCAGGGACA |  | *Cst* | | 58°C | |
| ***Hlx1.6-R*** | TAACAACTAAACAAAAGCCCAAA |  |  |  |  |  |
| ***Psmb9.8-F*** | TTCATTGTCCCCTTCCTGTA |  | *Cst* | | | 58°C |
| ***Psmb9.8-R*** | TACTGGGAAATGGCTGGTAA |  |  |  |  |  |
| ***Chr3 Cast1-F*** | TGCAAACGCTGGTGATGT |  | *Cst* | | | 58°C |
| ***Chr3 Cast1-R*** | AAGGACTTGGAGTGATGTGGA |  |  |  |  |  |
| ***Chr3 Cast2-F*** | ATCAGAATTGGTGGTCCTGAA |  | *Cst* | | | 62°C |
| ***Chr3 Cast2-R*** | ACTTGATCATGCAGCCTGTG |  |  |  |  |  |
| ***pSycp1-F*** | AAGACGACATGGAACGGAAC |  |  | | 56°C | |
| ***pSycp1-R*** | TTGAGCGCAGACTTGGTAGA |  |  |  |  |  |
